# FEAR OF FALLING IS RELATED TO ANXIETY

**DOI:** 10.1093/geroni/igac059.1886

**Published:** 2022-12-20

**Authors:** Hyeon Jung Kim, Jennifer Yentes, Dawn Venema, Julie Blaskewicz Boron

**Affiliations:** University of Nebraska Omaha, Omaha, Nebraska, United States; Texas A&M University, College Station, Texas, United States; University of Nebraska Medical Center, Omaha, Nebraska, United States; University of Nebraska, Omaha, Omaha, Nebraska, United States

## Abstract

Fear of falling (FOF), defined as a psychological symptom, is related to increased fall risk, anxiety, and depressive symptoms. As physical function changes with age, FOF can appear beginning in midlife. FOF may result in avoiding physical activities, resulting in weakness of postural balance. Neuroticism is associated with anxiety and depression; thus, those high on neuroticism may have an increased FOF. This study aimed to address the relationships between FOF, neuroticism, and number of falls from external perturbations while standing. Participants included 45 participants (female: n=24; age range: 42-77yrs; age=62.44±9.25yrs). Five standing trials were completed on a force platform with perturbations. Hierarchical regression was used to investigate the impact of neuroticism (Big Five Inventory) and number of falls on FOF (The Falls Efficacy Scale International), accounting for age, anxiety (Geriatric Anxiety Scale), and depressive symptoms (Center for Epidemiological Studies Depression Scale). Anxiety was significantly associated with FOF (model 1; age, anxiety, depression), B=0.31, β=.55, t(41)=3.08, R2=.30, p=.004, 95% CI [0.11, 0.51]. Neuroticism was not significantly related to FOF (model 2; ΔR2=.05, ΔF=2.94, p=.094), nor was number of falls (model 3; ΔR2=.00, ΔF=0.01, p=.974). Results revealed that anxiety levels had the strongest relationship with FOF. This suggests that strategies to reduce daily anxiety may decrease FOF. Future research should examine how anxiety is related to history of falls, and how changes in physical functions (e.g., mobility, vision, proprioception) may impact FOF.

